# Rare recessive loss-of-function methionyl-tRNA synthetase mutations presenting as a multi-organ phenotype

**DOI:** 10.1186/1471-2350-14-106

**Published:** 2013-10-08

**Authors:** Eline van Meel, Daniel J Wegner, Paul Cliften, Marcia C Willing, Frances V White, Stuart Kornfeld, F Sessions Cole

**Affiliations:** 1 Division of Hematology, Department of Internal Medicine, Washington University School of Medicine, St. Louis, MO 63110, USA; 2 Division of Newborn Medicine, Edward Mallinckrodt Department of Pediatrics, Washington University School of Medicine, St. Louis, MO 63110, USA; 3 Genome Technology Access Center, Department of Genetics, Washington University School of Medicine, St. Louis, MO 63110, USA; 4 Division of Genetics & Genomics Medicine, Edward Mallinckrodt Department of Pediatrics, Washington University School of Medicine, St. Louis, MO 63110, USA; 5 Department of Pathology and Immunology, Washington University School of Medicine, St. Louis, MO 63110, USA

**Keywords:** Methionyl-tRNA synthetase, Loss-of-function mutations, Aminoacylation

## Abstract

**Background:**

Methionyl-tRNA synthetase (MARS) catalyzes the ligation of methionine to its cognate transfer RNA and therefore plays an essential role in protein biosynthesis.

**Methods:**

We used exome sequencing, aminoacylation assays, homology modeling, and immuno-isolation of transfected MARS to identify and characterize mutations in the methionyl-tRNA synthetase gene (*MARS*) in an infant with an unexplained multi-organ phenotype.

**Results:**

We identified compound heterozygous mutations (F370L and I523T) in highly conserved regions of *MARS*. The parents were each heterozygous for one of the mutations. Aminoacylation assays documented that the F370L and I523T *MARS* mutants had 18 ± 6% and 16 ± 6%, respectively, of wild-type activity. Homology modeling of the human MARS sequence with the structure of E. coli *MARS* showed that the F370L and I523T mutations are in close proximity to each other, with residue I523 located in the methionine binding pocket. We found that the F370L and I523T mutations did not affect the association of MARS with the multisynthetase complex.

**Conclusion:**

This infant expands the catalogue of inherited human diseases caused by mutations in aminoacyl-tRNA synthetase genes.

## Background

Aminoacyl-tRNA synthetases (ARSs) are a family of enzymes that covalently attach transfer RNA (tRNA) and amino acids. The various ARSs function either in the cytoplasm or in mitochondria although in some cases they act in both compartments (i.e. bifunctional). Mutations in cytoplasmic and bifunctional ARSs have been identified in Charcot-Marie-Tooth disease (ARS for alanine (*AARS*), lysine (*KARS*), tyrosine (*YARS*) and glycine (*GARS*)) and distal spinal muscular atrophy type V (*GARS*) [1]. Interestingly, while these ARSs are ubiquitously expressed, these diseases are characterized by neurodegeneration and neuropathy. Also, cytoplasmic *DARS* mutations have been identified in recessive neurologic phenotypes [2] and *HARS* mutations in patients with peripheral neuropathy [3]. However, the disease phenotype associated with ARSs is expanding. For example, a recent report described a family kindred with infantile hepatopathy, anemia, renal tubulopathy, developmental delay, seizures and unusual fingers due to mutations in the gene that encodes cytoplasmic leucyl-tRNA synthetase (*LARS*) [4].

In this study we identified loss-of-function mutations in the gene encoding MARS, the cytoplasmic methionyl-tRNA synthetase, which couples methionine to tRNA, in an infant with a multi-organ phenotype similar to that observed in the patients with *LARS* mutations. The identified mutations significantly impaired MARS’ ability to ligate methionine to its cognate tRNA and are therefore likely responsible for the patient’s phenotype. This report provides additional evidence that mutations in cytoplasmic ARSs can lead to a variety of clinical manifestations beyond the nervous system.

### Case presentation

The female infant was the 2,500 g non-consanguineous product of a 36-week gestation in a 29-year-old primigravida woman. Paternal age was 29 years. Both parents were healthy without clinical evidence of neuropathy, and the family histories did not include first degree relatives with neurodegenerative or neuropathic syndromes or children with multi-organ failure. An evaluation was done at 1 month due to the failure to gain weight (60 g weight gain since birth) along with vomiting and mild hypotonia. The newborn screen was normal as were liver enzymes, but episodic hyperammonemia was noted along with anemia (hemoglobin 8.3 g%) with thrombocytosis (platelets 790,000/mm^3^) (Additional file 1: Table S1). An upper gastrointestinal series was normal.

**Figure 1:**
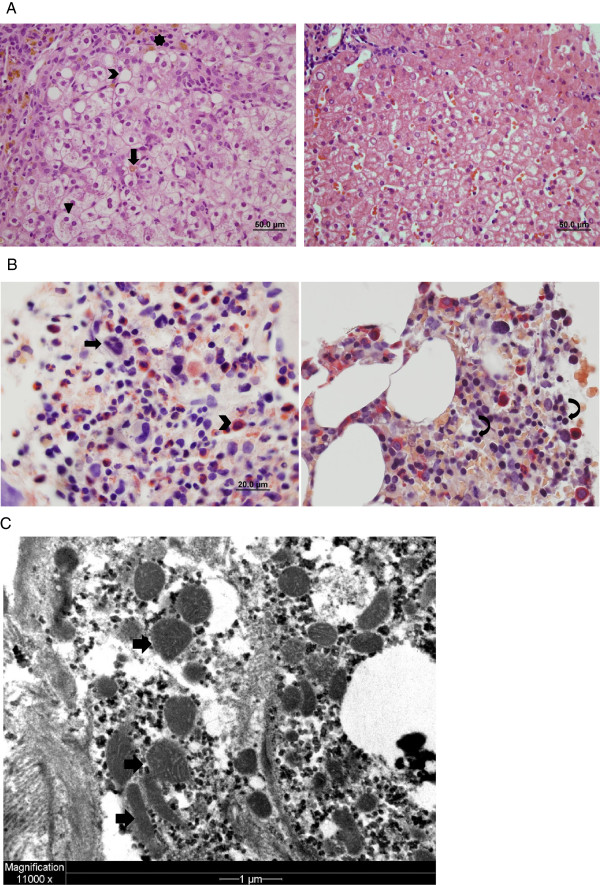
**Liver and bone marrow pathology. A**: The patient’s bone marrow (left photo) contains megakaryocytes (arrow) and numerous myeloid cells (chevron), while erythroid cells are difficult to identify. In contrast, erythroid cells (curved arrows) are readily apparent in control bone marrow (right photo). **B**: The patient’s liver (left photo) shows lobular disarray with hepatocyte ballooning (arrowhead), canalicular cholestasis (arrow) and zone 1 steatosis (chevron). Iron deposition (star) is present in macrophages and hepatocytes. Control liver (right photo) for comparison. **C**: Electron micrograph of hepatocyte shows mild pleomorphism of mitochondria (arrows), which contain predominantly flattened, straight and curved cristae. These are non-specific findings that can be seen in normal liver.

Between 3 and 9 months of age, the infant failed to gain weight (weight and head circumference less than 3^rd^ percentile) and developed liver failure, intermittent lactic acidosis, aminoaciduria, hypothyroidism, interstitial lung disease and transfusion-dependent anemia. Developmental delay (motor) and hypotonia were present, but MRI of the brain was normal. Bone marrow biopsy at 3 months showed arrest of RBC maturation (Figure 1A). Liver biopsy at 5 months revealed cholestasis, steatosis, bridging necrosis, minimal fibrosis, hemosiderin-laden macrophages in the portal tracts and normal appearing mitochondria (Figure 1B-C). Electron microscopy of the liver biopsy did not reveal diagnostic abnormalities (Figure 1C). Muscle biopsy revealed marked excess of type IIC muscle fiber consistent with mitochondrial disorders, but electron microscopic examination showed normal mitochondrial appearance. Succinate dehydrogenase and cytochrome C oxidase immunostaining in muscle was normal and genetic analysis excluded major mitochondrial rearrangements, including Pearson’s deletion, while DNA sequence analysis failed to identify pathogenic mutations in mitochondrial genes. Further, there was no evidence of a mitochondrial respiratory chain defect in muscle and liver tissues. Taken together these data excluded a primary mitochondrial disorder. Further evaluation excluded other known metabolic and genetic causes of this type of multi-organ phenotype (Table 1).

**Table 1:** **Diagnostic evaluation in a patient with *MARS *mutations**

**Disorders investigated**	**Investigations performed (normal unless otherwise indicated)**
**General investigations**	• Serology for Hepatitis B, C, EBV, CMV; PCR for HIV
	• Alpha-1-antitrypsin and Pi type
	• Sweat test
	• Chromosomal microarray analysis: Paternally inherited duplication of 3p14.2 (616 kb) including *C3orf67*
	• Immunoglobulin levels, neutrophil oxidative burst activity, lymphocyte subpopulations
**Disorders of intermediary metabolism**	• Glucose, creatine kinase
	• Serum amino acids including homocysteine
	• Urine organic acids (including succinylacetone)(during liver failure, tyrosine metabolites but not succinylacetone observed)
	• Serum ceruloplasmin, serum and urine copper
	• Serum iron profile: Iron 65 μg/dl (nl 50-120); iron binding capacity, unsaturated <20 μg/dl (nl 100-400), iron binding capacity total 40 μg/dl (nl 250-450), transferrin saturation 162% (nl 10-45); ferritin 1053 ng/ml (nl 50-200)
	• Galactosemia metabolic panel (GALT, Gal-1 PO4 levels and DNA testing)
	• Acylcarnitine profile
**Disorders of energy metabolism**	• Glucose profiling; lactate 3.4 mmol/L (nl 0.5-1.5), pyruvate 0.32 mmol/L (nl 0.03-0.08); CSF lactate 3.5, CSF pyruvate 0.19
	• Muscle biopsy: histology, histochemistry, electron microscopy, respiratory chain enzyme analysis, muscle mtDNA content, mtDNA analysis for deletions and rearrangements, mtDNA sequencing
	• Muscle biopsy: excess of type IIC muscle fibers, immunostaining for succinate dehydrogenase and cytochrome c oxidase normal, normal mitochondrial appearance by electron microscopy
	• *DGUOK*, *MPV17,* and *POLG1* sequencing
	• Ornithine decarbamylase gene sequencing
	• Liver biopsy: cholestasis, steatosis, bridging necrosis, minimal fibrosis, hemosiderin laden macrophages in the portal tracts, and normal appearing mitochondria and electron microscopy (Figure 1B-C), liver respiratory chain analysis, liver mtDNA content
	• Urine sugar and polyol and plasma sterol analyses
	• Initial aminoaciduria resolved
	• *BCS1L* sequencing for GRACILE (growth retardation, aminoaciduria, cholestasis, iron overload, lactacidosis, and early death) syndrome
	• Ophthalmology and cardiology assessments
	• MRI and CT scan of brain
**Disorders of complex molecules**	• Isoelectric focusing consistent with liver failure
	• White cell lysosomal enzyme screen
	• Wolman disease (lysosomal acid lipase deficiency)
	• Lysinuric protein intolerance (*SLC7A7*)
	• Urine mucopolysaccharides and oligosaccharides
	• Niemann-Pick types A and B (sphingomyelinase deficiency), and C (fibroblasts), GM1 gangliosidosis, and Gaucher disease
	• Bone marrow aspirate: RBC maturation arrest (precursors but not mature RBCs present); normal 5’nucleotidase
	• Plasma very long chain fatty acid analysis
	• Plasma and urine bile acid analysis, plasma cholesterol

Total parenteral nutrition (TPN) was started at age 4 months. At approximately 9 months of age the infant began to gradually improve becoming transfusion-independent and exhibiting normalization of liver function. However, at 3.5 years of age she remains dependent on TPN and nasal cannula oxygen, is less than 3^rd^ percentile for weight and head circumference, continues on thyroid replacement and has motor (but not cognitive) delay. Recent formal neurological assessment (age 3 years 9 months) including neurocognitive testing revealed motor delay and appropriate verbal and intellectual abilities. The patient has not undergone nerve conduction studies to assess her motor delay.

## Methods

### Antibodies and reagents

Rabbit anti-human MARS antibody (HPA004125) and rabbit anti-actin antibody (A2066) were obtained from Sigma (St. Louis, MO). Rabbit anti-human RARS antibody (ab31537) and rabbit anti-human KARS antibody (ab31532) were from Abcam (Cambridge, MA). Horseradish peroxidase linked donkey anti-rabbit IgG (GE Healthcare Biosciences, Pittsburgh, PA) was used as secondary antibody in western blotting. L-methionine [methyl-^3^H] with specific activity of 1.0 Ci/mmol was purchased from MP Biomedicals (Santa Ana, CA). Total tRNA type XI purified from bovine liver was from Sigma and calf liver tRNA from Novagen (kind gift from M. Boniecki/S. Martinis, University of Illinois, Urbana, IL). FLAG peptide was generated by Biomolecules Midwest Inc. (Waterloo, IL). All other reagents were from Sigma (St. Louis, MO), unless otherwise indicated.

### Exome sequencing

Genomic DNA was isolated from the patient’s blood using the Gentra Puregene blood kit (Qiagen, Germantown, MD) and from the parents’ saliva with Oragene collection kits (DNA Genotek Inc., Kanata, Canada). Exome targets were enriched with the SureSelect Human All Exon kit (38 MB version, Agilent Technologies, Santa Clara, CA), according to the manufacturer’s protocols and Illumina sequencing adapters (Illumina, Inc., San Diego, CA) were added to each sample to create sequence ready libraries. After sequencing with an Illumina HiSeq 2000 sequencing instrument the 2×101 bp paired-end sequencing reads were aligned to the human genome (reference build hg19) with Novoalign (Novocraft Technologies, Petaling Jaya, Malaysia) and genetic variants were identified with SAMtools [5]. Annovar [6] was used to annotate function of genetic variants and public databases (Exome Sequencing Project) to select rare variants (minor allele frequencies < 0.05). To predict deleterious effects of non-synonymous variants Annovar downloads data from dbNSFP [7], which recompiles prediction scores from four programs (SIFT, Polyphen2, LRT, and MutationTaster) as well as a conservation score (PhyloP). Species alignment was performed with Alamut (Interactive Biosoftware, San Diego, CA). Variant data for individuals of European descent (n = 4300) from the Exome Sequencing Project were annotated and selected in the same manner as the patient’s and parents’ exome sequencing data and used to estimate potential disease prevalence by calculating a collapsed, deleterious, non-synonymous *MARS* allele frequency.

### Constructs

Human *MARS* cDNA (NM_004990.3) in pCMV6-AC was obtained from OriGene Technologies, Inc. (Rockville, MD). MARS mutants F370L and I523T were generated by quick change mutagenesis, using the primers 5′-CCA AAA TCA CCC AGG ACA TTC TCC AGC AGT TGC TGA AAC G-3′ and 5′-CGT TTC AGC AAC TGC TGG AGA ATG TCC TGG GTG ATT TTG G-3′ for F370L MARS and the primers 5′-CTG GTT TGA TGC CAC TAC TGG CTA TCT GTC CAT C-3′ and 5′-GAT GGA CAG ATA GCC AGT AGT GGC ATC AAA CCA G-3′ for I523T MARS. For purification, a C-terminal FLAG sequence was introduced by PCR with the following primers 5′-CCG CTC GAG GCC ACC ATG AGA CTG TTC GTG AGT G-3′ and 5′-CCC AAG CTT TTA CTT GTC ATC GTC GTC CTT GTA GTC CTT TTT CTT CTT GCC-3′. Wild-type, F370L or I523T *MARS*-FLAG was excised with XhoI and HindIII and inserted into pcDNA3.1(−).

### Aminoacylation assay

HEK293 cells were cultured in 6-well plates in Dulbecco’s Modification of Eagle’s Medium (DMEM, Cellgro, Mediatech, Inc., Manassas, VA) containing 4.5 g/L glucose with 10% fetal bovine serum (Atlanta Biologicals, Lawrenceville, GA), L-glutamine (Cellgro) and penicillin/streptomycin (Invitrogen, Carlsbad, CA). The cells were transfected with pCMV6-AC that encoded wild-type, F370L or I523T MARS, or mock transfected using Lipofectamine Plus reagent (Invitrogen) according to the manufacturer’s protocol. Nineteen hours after transfection the cells were lysed in 1% Triton-X100 in 25 mM HEPES, pH = 7.9, containing 10% glycerol and a protease inhibitor cocktail (Complete, Roche Diagnostics, Indianapolis, IN). The protein concentration was determined via the Bradford protein assay and 20 μg was added to the reaction mixture, which contained 25 mM HEPES, 3 mM MgCl_2_, 2 mM DTT, 2 mM ATP, 160 μg tRNA, 1 μCi of [^3^H]-methionine in a final concentration of 50 μM, 10% glycerol, pH = 7.9, in a total volume of 100 μL. The reactions were incubated at room temperature for 10 minutes (time course studies showed that the reaction was still linear at this incubation period), after which the reaction mixture was spotted on filter pads (Whatman, grade 3, GE Healthcare UK Limited, Little Chalfont, UK) that had been pre-wetted in 5% trichloroacetic acid and dried. The filter pads were washed 3 times in 5% trichloroacetic acid, once in 70% ethanol and subsequently dried and counted in a scintillation counter. The values obtained with mock transfected cell lysates were subtracted from the values obtained with wild-type or mutant MARS expressing cells to correct for endogenous enzyme activity and non-specific background counts. MARS expression in HEK293 lysates was verified by subjecting 10 μg of lysate to SDS-PAGE and western blotting.

### MARS purification

Wild-type, F370L or I523T MARS-FLAG cDNAs in pcDNA3.1(−) were expressed in HEK293 cells as described. After lysis in 1% Triton-X100/25 mM HEPES, pH = 7.9/10% glycerol containing protease inhibitors, the lysates were incubated with anti-FLAG M2 affinity gel for 2 h at 4°C, while rotating. Subsequently, the beads were washed with cold PBS and bound protein was eluted from the beads by incubation with 0.5 mg/ml FLAG peptide in 25 mM HEPES/10% glycerol at 4°C on a rotor. After 45 minutes the beads were spun down and the supernatant, containing FLAG-tagged MARS, was collected. The eluted fractions were separated by SDS-PAGE, and proteins were detected with Coomassie brilliant blue.

## Results

### Genetic analysis

**Figure 2:**
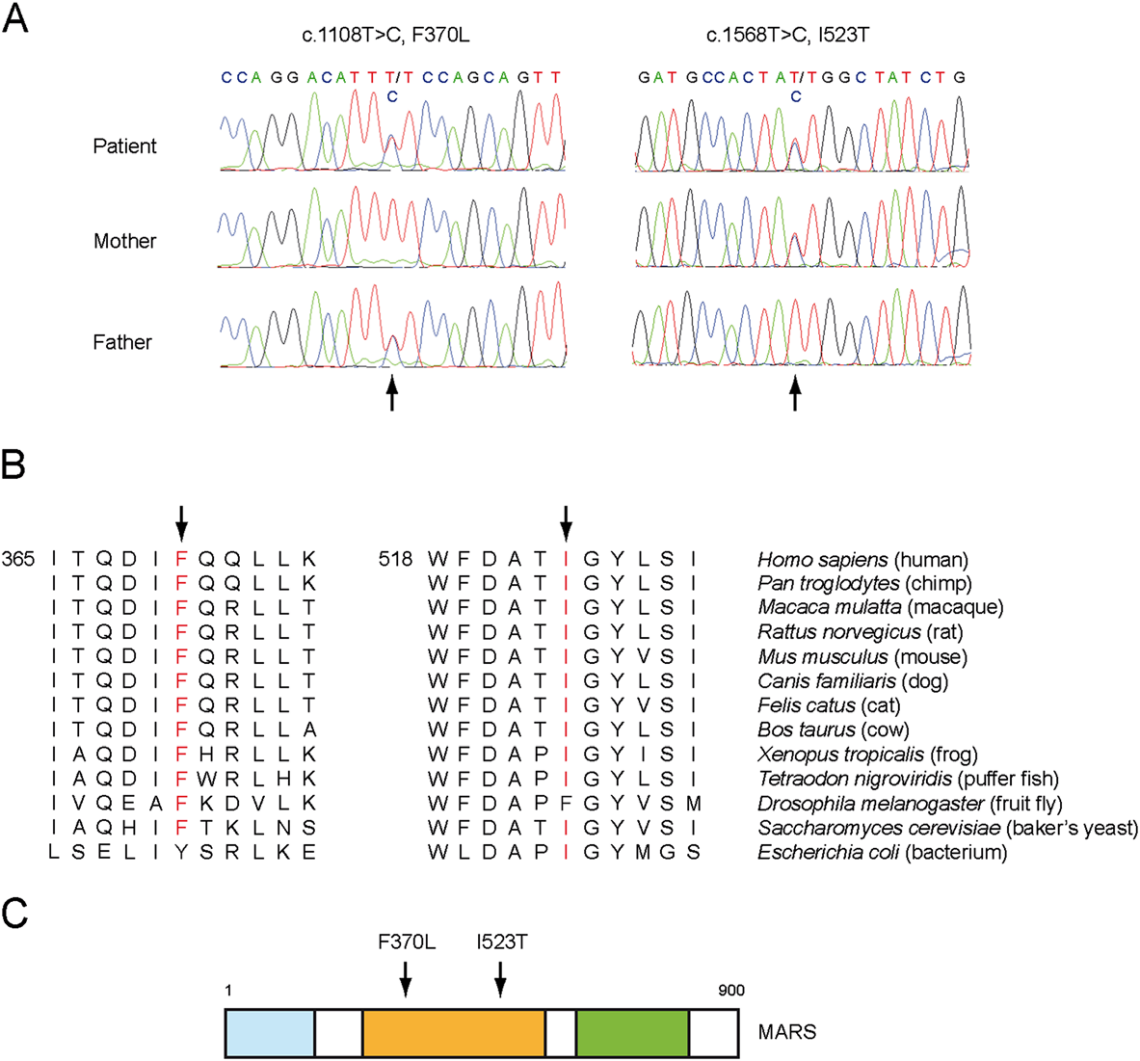
**Exome sequencing reveals mutations in highly conserved regions of the catalytic domain of MARS. A**: *MARS* mutations in the patient and her parents. The patient is compound heterozygous for the mutations c.1108 T > C, F370L (paternally inherited) and c.1568 T > C, I523T (maternally inherited), see arrows. **B**: Protein sequence alignment of MARS orthologs. The mutated residues are indicated by arrows and marked in red when conserved. **C**: Schematic representation of the different domains of MARS. The locations of the mutations, which are in the catalytic domain (orange), are indicated by arrows. The GST-like domain is shown in blue and the tRNA binding domain in green.

Chromosomal microarray analysis revealed a 616 kb duplication of chromosome 3 (3p14.2) (data not shown). This region contains only one gene (*C3orf67*). Since the father had the same duplication and was asymptomatic and the mother had no evidence of disruptive variants in this region, we reasoned that the duplication was unlikely to be responsible for this patient’s disorder. Therefore, to identify candidate gene mutations, we sequenced the exomes of the patient and her parents. In total, 17,988 variants (single nucleotide polymorphisms and small insertions/deletions) were identified in the patient, and various computational filtering criteria were used to select candidate genes. Recessive inheritance of rare mutations was assumed, and variants that were non-exonic, synonymous or present at frequencies ≥ 0.05 in the Exome Sequencing Project database were excluded. Furthermore, only non-synonymous variants predicted to be functional by at least 4 of the 5 functional prediction algorithms in Annovar [6] were selected. Finally, only genes were selected in which multiple loss of function alleles were present. Genes with *de novo* mutations were also included. This filtering strategy resulted in the identification of a single gene, *MARS* (NM_004990.3), which encodes methionyl-tRNA synthetase. The patient was compound heterozygous for two missense mutations, c.1108 T > C in exon 10, resulting in F370L and c.1568 T > C in exon 13, changing Ile at 523 to Thr (Figure 2A). Each parent was heterozygous for one of the mutations; the father carried c.1108 T > C, and the mother c.1568 T > C (Figure 2A). Sanger sequencing confirmed both mutations in the patient and her parents.

### Effect of mutations on MARS expression and activity

Alignment of human MARS with MARS from different species showed that both mutated residues are located in highly conserved regions (Figure 2B). Phenylalanine at position 370 in human MARS was conserved in all species that were analyzed with the exception of *E. coli*, which has a similar hydrophobic residue, i.e. tyrosine. Isoleucine at position 523 was conserved in all species except in *Drosophila melanogaster*, which has phenylalanine at this position (Figure 2B). Moreover, both mutations are present in the catalytic domain of MARS (Figure 2C) and therefore were likely to have an effect on MARS aminoacylation function.

**Figure 3:**
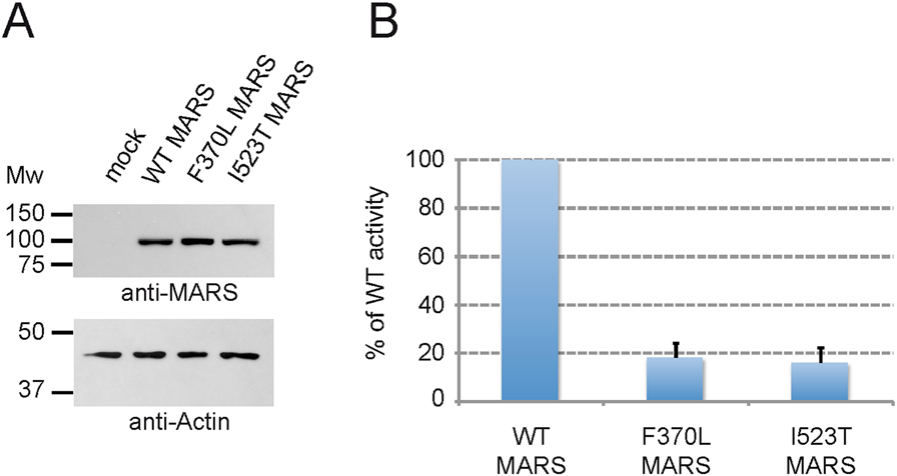
**MARS mutations F370L and I523T significantly impair MARS activity. A**: F370L and I523T MARS are expressed in HEK293 cells. Western blots show that wild-type and the mutant forms of MARS were expressed at similar levels (upper panel) with equal amounts of protein loaded, as reflected by the equal levels of actin (lower panel). Endogenous MARS in mock treated cells was only detected after prolonged exposure (see Additional file 2: Figure S1). **B**: Measurement of MARS activity, using the aminoacylation assay, shows greatly reduced activity of both *MARS* mutants. The activities of F370L and I523T MARS are represented as the percentages of wild-type MARS activity (set to 100%). The values are the averages of 3 independent experiments, ± the standard deviations.

To determine whether the mutations impaired MARS activity we measured the ability of the various forms of MARS to couple [^3^H]-methionine to tRNA in an *in vitro* aminoacylation reaction. We initially assayed extracts of patient and control fibroblasts, but the MARS activity in these samples was too low to obtain valid data. For this reason, we generated plasmids encoding human wild-type, F370L or I523T MARS and used these to transfect HEK293 cells. After 16 h, cell lysates were examined for MARS expression by western blotting. Similar levels of wild-type, F370L and I523T MARS were detected (Figure 3A; Additional file 2: Figure S1), indicating that the *MARS* mutations did not impair expression or half-life of the enzyme. Whole cell lysates with equal amounts of MARS were then used in the aminoacylation assay. Both mutants had detectable, but low levels of activity compared to wild-type MARS. The F370L mutant had 18 ± 6% and the I523T mutant had 16 ± 6% of wild-type activity (Figure 3B), demonstrating that each mutation significantly reduces the ability of MARS to couple methionine to its cognate tRNA.

**Figure 4:**
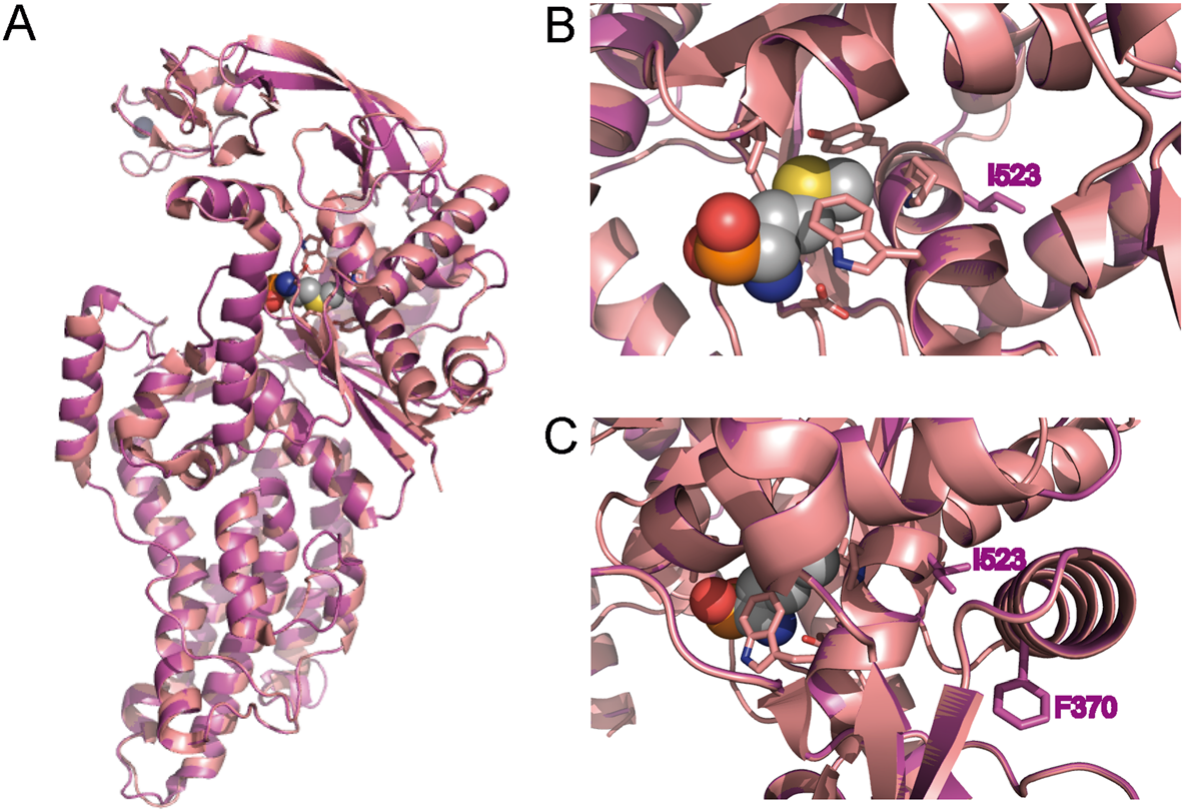
**Location of the mutated residues I523 and F370 in human MARS. A**: Homology modeling of human MARS (purple) with *E. coli* MARS (pink, PDB 1PFU) in complex with methionine phosphinate (grey, with elements colored in red (oxygen), orange (phosphorus), yellow (sulfur) and blue (nitrogen)). **B**: Close-up of the methionine binding pocket. Residues that interact with methionine are represented by sticks (pink). Residue I523 of human MARS (purple sticks) is located close to the methionine binding pocket, but does not directly interact with methionine. **C**: Residue F370 (purple sticks) is located in an alpha-helix close to residue I523.

To gain more insight into the consequences of the mutations, human MARS was modeled on the structure of *E. coli* MARS [8], which shares 28% identity and 48% similarity with the human MARS sequence (Figure 4A). This homology modeling revealed that residue I523 is located in an alpha-helix that is part of the methionine binding pocket that contains several residues that directly interact with methionine (Figure 4B). While I523 is not directly involved in methionine binding, its close proximity to residues that are critical for this binding suggests that the mutation could affect this interaction. The presence of F370 in an alpha-helix close to I523 (Figure 4C) could explain the effect of mutation of this residue on MARS activity.

### *MARS* mutations do not impair multisynthetase complex assembly

**Figure 5:**
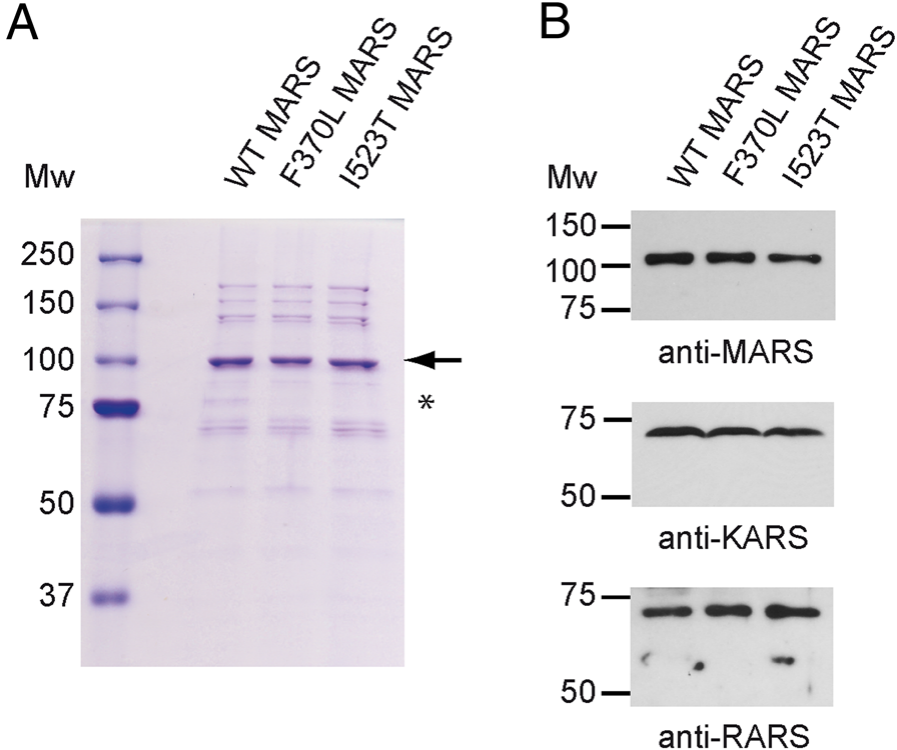
**F370L and I523T MARS associate with the multisynthetase complex. A**: Coomassie staining of wild-type, F370L and I523T MARS-FLAG purified fractions. MARS-FLAG is indicated (arrow). The asterisk shows a degradation product of MARS (~75 kD), which is more pronounced after purification of wild-type MARS-FLAG. However, the ratio of the 100 kD full length MARS and the partially degraded form was not established in the wild-type and *MARS* mutants. MARS was not immunoprecipitated from cell lysates that expressed I523T MARS-FLAG with a nonsense mutation in the FLAG tag that abolished binding to the anti-FLAG antibody (see Additional file 3: Figure S2). **B**: Components of the multisynthetase complex are co-purified upon immuno-isolation of wild-type, F370L or I523T MARS. Western blotting shows the presence of similar levels of MARS, KARS and RARS (upper, middle and lower panel, respectively) after immuno-isolation of MARS-FLAG from HEK293 cells.

MARS, along with eight other synthetases and three non-enzymatic cofactors, is known to form a multisynthetase complex [1]. While the role of the complex has remained elusive, several potential functions have been proposed [9]. It was therefore of interest to determine whether the mutations in *MARS* altered its incorporation into the complex. To do this, FLAG-tagged wild-type, F370L and I523T MARS cDNAs were generated and expressed in HEK293 cells. Subsequently, MARS was immuno-isolated, and the co-purified proteins were visualized by Coomassie staining (Figure 5A; Additional file 3: Figure S2). In all three instances, a staining pattern consistent with the multisynthetase complex was observed [10]. Western blotting identified two ARSs that are known components of the complex, i.e. lysyl-tRNA synthetase (KARS) and arginyl-tRNA synthetase (RARS) (Figure 5B). These data show that while the F370L and I523T mutations impair MARS activity, they do not prevent its association with the multisynthetase complex.

## Discussion

In this study, we identified two mutations in the gene encoding MARS in an infant with an unexplained disorder. Since each mutation significantly reduces MARS activity, they are likely responsible for the multi-organ phenotype observed in the patient. The lack of symptoms in her heterozygous parents indicates that a predicted ~40% reduction of MARS activity is well tolerated. While the mutations impair the catalytic activity, they do not affect the association of MARS with the multisynthetase complex. This finding is in agreement with a previous study, which showed that the N-terminal GST-like subdomain of MARS is required for its association with the complex [11]. Since the role of the multisynthetase complex is unclear, it is not possible to know at this point whether the *MARS* mutations have any negative consequences on the function(s) of the complex. In addition, a paternally inherited 616 kb duplication of 3p14.2 was observed in the patient. Although the father was unaffected and no maternal potentially trans-acting mutations were noted in the region of this duplication, we cannot exclude the possibility that the duplication contributes in some way to the patient’s phenotype.

Due to the unique characteristics of this patient’s phenotype, searching for other patients with *MARS* mutations and similar phenotypes is difficult. To attempt to estimate disease prevalence due to *MARS* mutations we used variant data from the Exome Sequencing Project (Exome Variant Server, NHLBI GO Exome Sequencing Project (ESP), Seattle, WA (URL: http://evs.gs.washington.edu/EVS/; n = 4,300 European descent individuals) (Downloaded August 20, 2012). After variant filtering, we identified 33 predicted loss-of-function alleles. Assuming Annovar accurately predicts loss of function, and that mutations are not embryonic or fetal lethal or associated with variable phenotypes, we estimated a maximum disease frequency associated with loss of *MARS* function at 1.5 /100,000 individuals, or approximately 33 cases/year from an annual U.S. European descent birth cohort of approximately 2,200,000 [12]. Confirmation of causality by discovery of *MARS* mutations in a cohort of patients with similar, rare phenotypes was not possible.

Surprisingly, the condition of the patient began to improve at approximately 9 months of age. While the explanation for her improvement is unclear, we considered the possibility that the initiation of parenteral nutrition may have contributed to the improvement. Since the mutated residues present in the patient’s MARS are close to the methionine binding pocket, we hypothesized that the mutations might impair methionine binding. In that scenario, increased methionine intake via parenteral nutrition might improve MARS aminoacylation function. However, when we tested this hypothesis in fibroblasts obtained from the patient, we were unable to find any clear indication that their slow growth rate was altered in the presence of higher concentrations of methionine (data not shown).

Based on the observation that mutations in cytoplasmic ARSs result in neurologic phenotypes, it was postulated that ARSs may be particularly important for the development and function of neurons, and that mutations in all ARSs may result in neurological disease [1]. However, more recently it has become clear that mutations in mitochondrial ARSs result in more heterogeneous phenotypes (*SARS2*, *AARS2*, *HARS2, LARS2* and *YARS2* mutations) [13-17] that may include neurologic phenotypes (*MARS2, RARS2, FARS2, DARS2* and *EARS2*) [18-23]. Interestingly, our patient’s phenotype has characteristics similar to the phenotype recently observed in an Irish Traveller family kindred characterized by hepatopathy in the first 6 months of life and associated with homozygous, novel or rare, loss-of-function mutations in the gene that encodes LARS [4]. Similar to our *MARS* mutation patient, the *LARS* patients exhibited anemia, renal tubulopathy, developmental delay, failure to thrive, hepatopathy and unusual fingers. However, our patient has distinct differences from the *LARS* patients including interstitial lung disease, hypothyroidism and normal brain MRI. These phenotypic differences may be attributable to differences in requirement for the two ARSs, different levels of residual activity or genetic background differences. Interestingly, among patients with autoimmune antisynthetase syndromes, antibodies against aminoacyl-tRNA synthetases strongly predict interstitial lung disease [24-26]. Our patient’s interstitial lung disease may reflect a mechanistically distinct but functionally similar disruption of MARS.

## Conclusions

The current study shows that loss-of-function of MARS, a cytoplasmic ARS, causes a multi-organ disorder with greater impact on liver, bone marrow, lung and thyroid function than on neuronal function during infancy.

After submission of this manuscript, Gonzalez *et al.* reported two male family members with late onset Charcot-Marie-Tooth disease who are heterozygous for a R618C missense mutation in MARS [27]. The Arg618 residue is located in the catalytic domain of the enzyme and the Cys substitution results in impaired activity as measured in a yeast rescue assay. In view of this report, regular surveillance for neuropathy in this patient and the parents will be important.

### Consent

Written informed consent was obtained from both parents for exome sequencing and publication of this Case Report and any accompanying images. A copy of the written consent is available for review by the Editor of this journal.

## Abbreviations

tRNA: Transfer RNA; MARS: Methionyl-tRNA synthetase; MARS: Methionyl-tRNA synthetase gene; ARS: Aminoacyl-tRNA synthetase.

## Competing interests

The authors declare that they have no competing interests.

## Authors’ contributions

EvM performed the characterization of the *MARS* mutations and drafted the manuscript. DJW performed exome sequencing and computational analysis of sequence data. PC performed computational analysis of sequence data. MCW clinically characterized the patient and drafted the patient summary. FW characterized and described the patient’s pathological samples. SK conceived of the study, participated in its design, and helped to draft the manuscript. FSC participated in the design of the study and helped to draft the manuscript. All authors read and approved the final manuscript.

## Pre-publication history

The pre-publication history for this paper can be accessed here:

http://www.biomedcentral.com/1471-2350/14/106/prepub

## Supplementary Material

Additional file 1: Table S1 Multi-organ dysfunction in a patient with *MARS* mutations. Click here for file

Additional file 2: Figure S1 Endogenous MARS is detected in HEK293 cells. HEK293 cell lysates that were mock transfected or transfected with wild-type, F370L or I523T MARS were subjected to SDS-PAGE and anti-MARS western blotting. Prolonged exposure shows the presence of endogenous MARS in the mock transfected cells. Click here for file

Additional file 3: Figure S2 F370L and I523T MARS associate with the multisynthetase complex. As a control, HEK293 cells were transfected with a construct encoding I523T MARS with a mutation in the FLAG-tag that abolished binding to the anti-FLAG antibody. The anti-FLAG immunoprecipitates of lysates of these cells showed two Coomassie staining background bands (arrows), but lacked the MARS band and the other components of the complex. Click here for file
