# Supplementary material for: Rare recessive loss-of-function methionyl-tRNA synthetase mutations presenting as a multi-organ phenotype
Source: BMC Med Genet. 2013 Oct 8;14:106. doi: 10.1186/1471-2350-14-106 (PMC3852179; doi:10.1186/1471-2350-14-106)
Supplement: Additional file 1: Table S1 — Multi-organ dysfunction in a patient with MARS mutations. [file 1471-2350-14-106-S1.doc]

**Table S1 – Multi-Organ Dysfunction in a Patient with *MARS* Mutations**

|  | **Organ System** | | | | | | | | **Summary** |
| --- | --- | --- | --- | --- | --- | --- | --- | --- | --- |
| **Age** | **GI** | **NEURO** | **HEM** | **ENDO** | **RENAL** | **PUL** | **DEV** | **MUSC** |  |
| **1 MO** | **+** | **+** | **+** |  |  |  |  |  | Normal newborn screen; failure to gain weight; episodic hyperammonemia; vomiting; hypotonia; mild anemia; thrombocytosis |
| **3 MO** | **++** | **+** | **+** |  |  |  | **+** |  | FTT; progressive hepatomegaly and liver failure (transaminitis, [hyperammonemia](http://emedicine.medscape.com/article/1174503-overview), hyperbilirubinemia) with coagulopathy, hypoalbuminemia (<1 g%); hypotonia; anemia (bone marrow – arrest in RBC maturation); thrombocytopenia; developmental delay (motor) |
| **6 MO** | **++** | **+** | **++** | **+** | **+** | **+** | **+** |  | FTT (TPN-dependent); hepatomegaly and liver failure (transaminitis, hyperammonia, hyperbilirubinemia); hypotonia; transfusion-dependent anemia; thrombocytopenia; hypothyroidism; proximal renal tubular dysfunction (non-specific aminoaciduria); interstitial lung disease; developmental delay (motor) |
| **8 MO** | **+** | **+** | **++** | **+** | **+** | **+** | **+** | **+** | FTT (TPN-dependent); liver dysfunction (transaminitis, hypoalbumenia); hypotonia (normal brain MRI); transfusion-dependent anemia; hypothyroidism; aminoaciduria; interstitial lung disease; developmental delay (motor); osteopenia |
| **10 MO** | **+** | **+** | **+** | **+** |  | **+** | **+** | **+** | FTT (TPN-dependent); liver dysfunction; non-transfusion-dependent anemia; hypothyroidism; interstitial lung disease; developmental delay (motor); osteopenia |
| **12 MO** | **+** | **+** | **+** | **+** |  | **+** | **+** | **+** | FTT (TPN-dependent); liver dysfunction (transaminitis, hypoalbuminemia); hypotonia; mild anemia; hypothyroidism; interstitial lung disease; developmental delay (motor); osteopenia |
| **24 MO** | **+** | **+** | **+** | **+** |  | **+** | **+** |  | FTT (TPN-dependent); liver dysfunction; hypotonia; mild anemia; hypothyroidism; interstitial lung disease; motor development and cognition improving |
| **36 MO** | **+** | **+** | **+** | **+** |  | **+** | **+** |  | FTT (TPN-dependent); liver dysfunction improved; hypotonia; mild anemia with thrombocytopenia; interstitial lung disease; motor development and cognition improving |

FTT – failure to thrive (head circumference, length, and weight <3^rd^ percentile); RBC – red blood cell; TPN – total parenteral nutrition
