# Supplementary figures and images for: Rare recessive loss-of-function methionyl-tRNA synthetase mutations presenting as a multi-organ phenotype
Source: BMC Med Genet. 2013 Oct 8;14:106. doi: 10.1186/1471-2350-14-106 (PMC3852179; doi:10.1186/1471-2350-14-106)

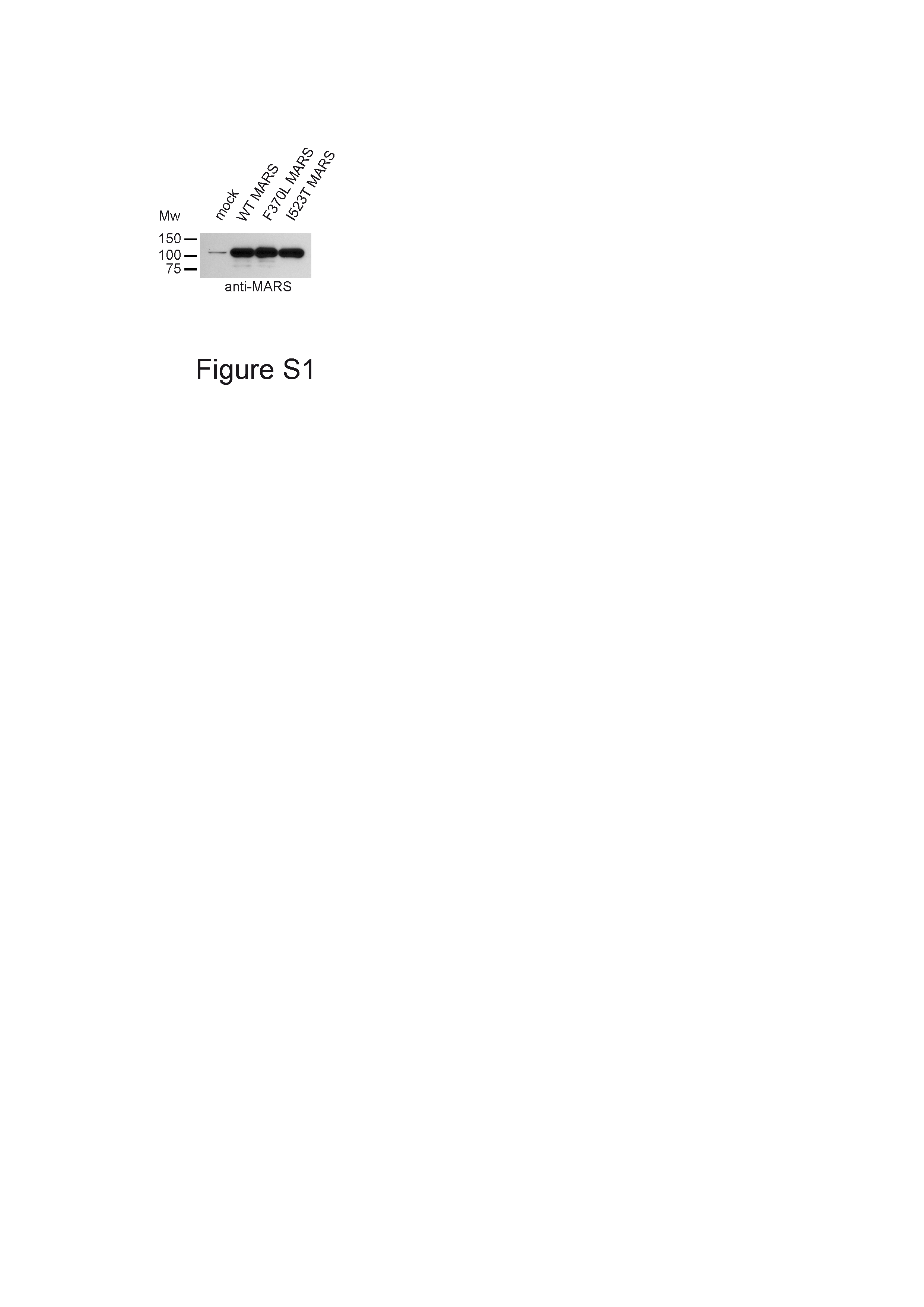

Supplement: Additional file 2: Figure S1 — Endogenous MARS is detected in HEK293 cells. HEK293 cell lysates that were mock transfected or transfected with wild-type, F370L or I523T MARS were subjected to SDS-PAGE and anti-MARS western blotting. Prolonged exposure shows the presence of endogenous MARS in the mock transfected cells. [file 1471-2350-14-106-S2.tiff]

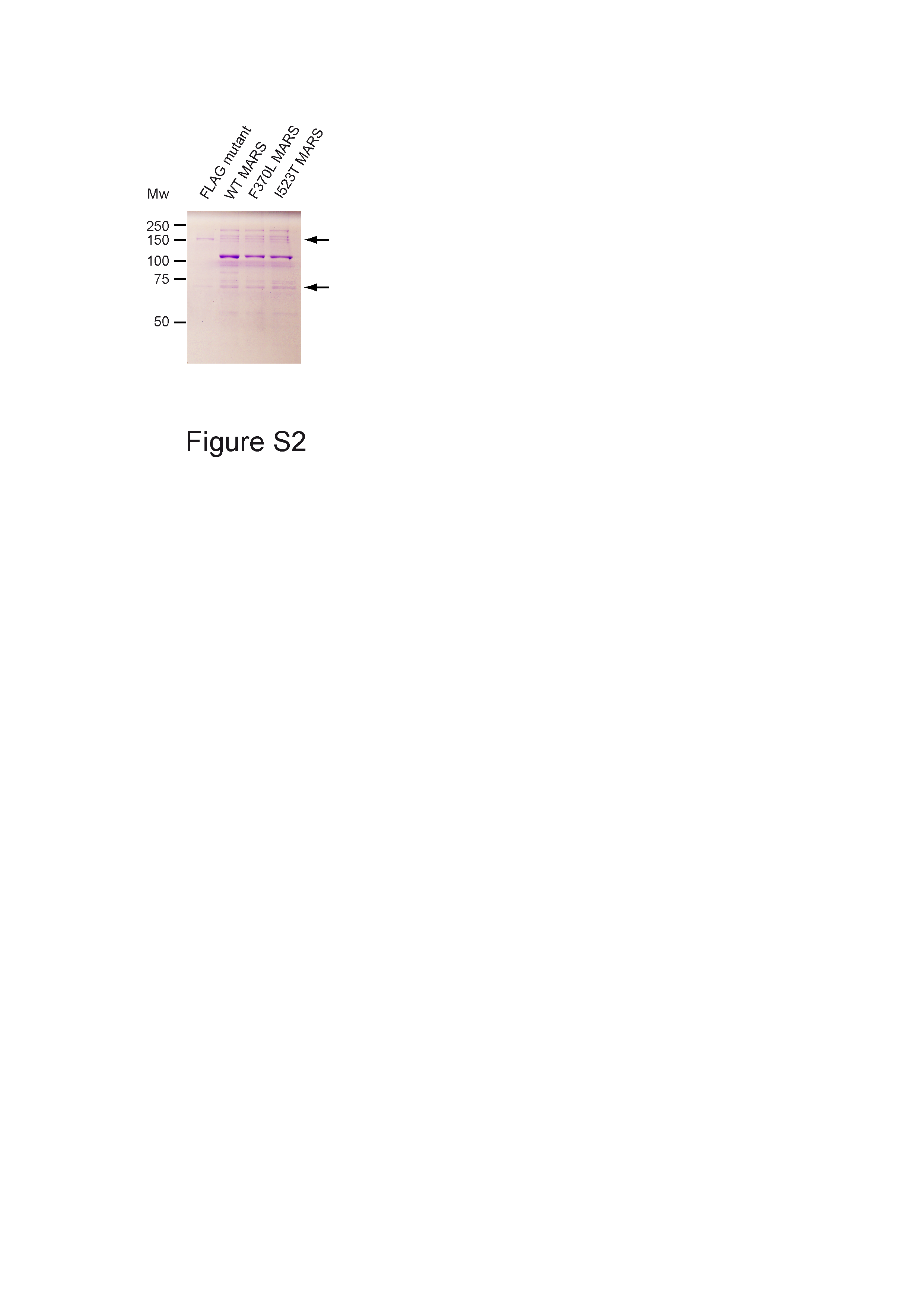

Supplement: Additional file 3: Figure S2 — F370L and I523T MARS associate with the multisynthetase complex. As a control, HEK293 cells were transfected with a construct encoding I523T MARS with a mutation in the FLAG-tag that abolished binding to the anti-FLAG antibody. The anti-FLAG immunoprecipitates of lysates of these cells showed two Coomassie staining background bands (arrows), but lacked the MARS band and the other components of the complex. [file 1471-2350-14-106-S3.tiff]
